# Transomental defects as a cause of chronic abdominal pain, the role of diagnostic laparoscopy: a case series

**DOI:** 10.4076/1757-1626-2-8356

**Published:** 2009-07-06

**Authors:** Arun V Ariyarathenam, Tjun Y Tang, Senthil Nachimuthu, Yashwant Koak, Adrian M Harris

**Affiliations:** Department of General Surgery, Hinchingbrooke NHS Trust HospitalHuntingdon, PE29 6NTUK

## Abstract

**Introduction:**

Transomental herniation is a rare but recognised clinical condition, which usually presents as an emergency with bowel obstruction. It accounts for 1-4% of intra-abdominal herniations. We reviewed 3 patients found to have a transomental defect during elective diagnostic laparoscopy performed for chronic abdominal pain. To our knowledge, there is no case series reported in the literature on transomental defect in the non-emergency situation.

**Case presentation:**

A retrospective case note analysis of 3 patients, found to have transomental defect during elective diagnostic laparoscopy, was undertaken. Data were gathered with respect to clinical presentation, investigations performed, transomental defect size and outcome of surgery. All patients were followed up for 6 months post-operatively. Three females (age range 18-35 years) were referred with a 3-10 year history of chronic intermittent abdominal pain, often postprandial. Blood tests, radiological investigations (ultrasound, magnetic resonance imaging/computed tomography, small bowel studies) and endoscopy were all normal. In each case, diagnostic laparoscopy revealed the presence of a peripheral defect in the greater omentum, but no actual small bowel herniation. No other pathology was found. These defects were resected, which subsequently led to complete resolution of the patients’ symptoms.

**Conclusion:**

Chronic abdominal pain of unknown aetiology with normal radiological findings may be caused by intermittent obstruction due to small bowel herniation through a transomental defect. This should be considered during elective diagnostic laparoscopy, in the absence of any other obvious pathology. The omentum should be thoroughly inspected as a discrete entity and any such defects should be closed or resected.

## Introduction

Transomental hernias are rare accounting for 1-4% of all internal hernias [1,2]. The majority are through defects in the greater omentum and very rarely through lesser omental defects. These hernias usually present acutely with abdominal pain and intestinal obstruction. The diagnosis is often made at the time of surgery or through radiological investigations, such as CT or Barium studies [3-5].

The aim of this study was to document the number of patients where transomental defects (TOD) were found in patients undergoing elective diagnostic laparoscopy for chronic abdominal pain and whether the correction of the defect led to resolution of the symptoms.

## Case presentation

A retrospective analysis of 3 patients with TOD found at diagnostic laparoscopy was undertaken. Our initial case led to the finding of a large congenital TOD, which led to the hypothesis that it may have been responsible for intermittent transomental herniation of likely small bowel and thereby explaining the patient’s symptoms. The correction of the defect led to complete resolution of the symptoms immediately after surgery and this was confirmed at her 6 months follow-up. This led us to scrutinise the greater omentum more closely during similar cases, leading to two further cases with similar findings.

### Case 1

An 18-year-old British Caucasian female presented to the gastroenterology team with a 5-year history of intermittent abdominal pain. She has always been a very active individual both in sports and education, but was affected by sudden onset central cramping abdominal pain, often postprandial followed by episodes of diarrhoea. The pain would resolve spontaneously. Neither the frequency nor the duration was predictable.

Her haematological & biochemical results were normal. Radiological investigations in the form of ultrasound, magnetic resonance cholangiopancreatography (MRCP) and small bowel follow through were also within normal limits. The patient’s only past medical history included an open appendicectomy at the age of 5 years for a non-perforated inflamed appendicitis.

**Figure 1.**
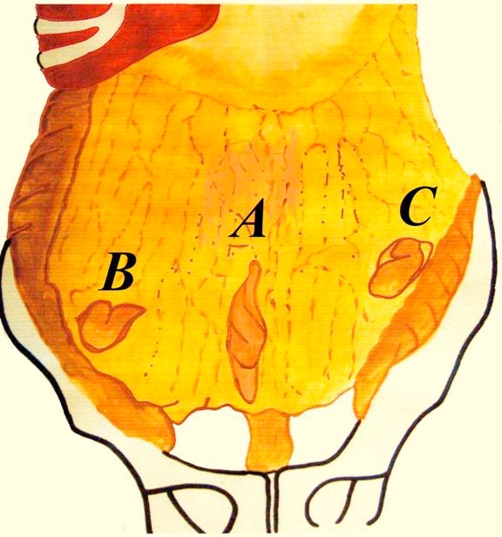
Diagram summarising the positions of the transomental defects in the cases.

**Figure 2.**
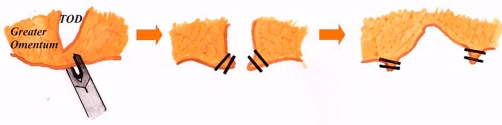
Diagram showing resecting open of the transomental defect between clips performed at laparoscopy.

The general surgical team performed a diagnostic laparoscopy in hope of finding a diagnosis. This revealed a large central 5 cm × 6 cm greater omental defect on the inferior border (Figure 1A). The omental defect was resected open by dividing the inferior portion on the omentum forming the defect and the mesenteric vessels controlled with haemostatic clips (Figure 2). No other findings were found at the time. Complete resolution of symptoms was reported by the patient at 6 months follow-up.

### Case 2

A 35-year-old British Caucasian female was managed under the care of gastroenterology, paediatrics & gynaecology with a 10-year history of chronic right iliac fossa pain. Her endoscopic examinations (OGD & colonoscopy) and radiological investigations (ultrasound of abdomen and pelvis, barium small bowel meal, MRCP & CT (abdomen and pelvis) were normal. Her only significant past medical history included a diagnostic laparoscopy by the gynaecologist when she was 25 for mid-cycle pain with associated dyspareunia. There were no positive findings at that laparoscopy and her gynaecological symptoms settled, but she continued to have persistent right iliac fossa pain, which was colicky in nature with associated nausea and vomiting and settle spontaneously with pain free episodes.

**Figure 3.**
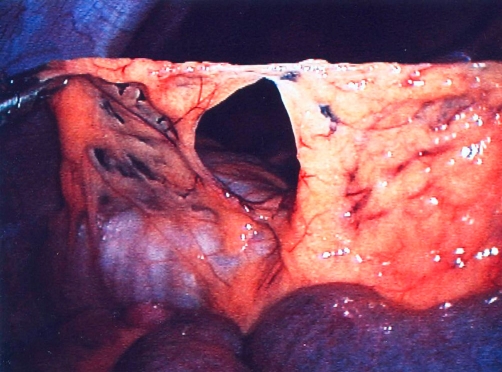
Laparoscopic image of the transomental defect of Case 2.

Repeat diagnostic laparoscopy revealed a macroscopically normal looking appendix and an omental adhesion forming a 3 × 4 cm defect (Figure 1B, 3). The adhesion was divided and the omental defect corrected. She subsequently made a good post-operative recovery with resolution of her symptoms at 6 months.

### Case 3

**Figure 4.**
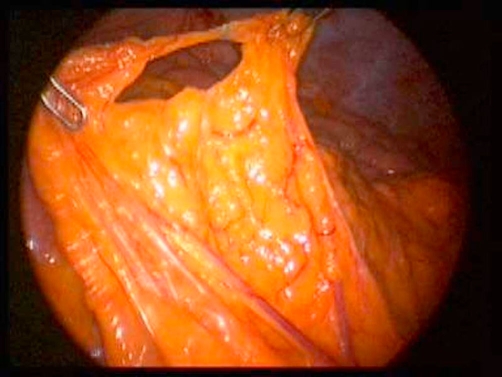
Laparoscopic image of the transomental defect of Case 3.

A 28-year-old British Caucasian female presented with a 3-year history of chronic abdominal pain. The pain was described as being postprandial but with no correlation with food types. It was mainly central in site but often generalised. OGD & colonoscopy with biopsies, CT abdomen and pelvis, small bowel follow through and ultrasound abdomen and pelvis were normal. Diagnostic laparoscopy revealed the presences of a 2 cm × 3 cm greater omental defect on the left margin (Figure 1C, 4). The omental defect was resected and this led to resolution of her symptoms at 6 months follow up.

## Discussion

TOD are often recognised when patients present with acute intestinal obstruction mainly involving the small bowel but also segments of the large bowel such as sigmoid and caecum. These can sometimes be confirmed using pre-operative radiology but often at the time of emergency surgery.

In our small but yet interesting case series, the patients were younger than the usual age of greater than 50 years [5], with the oldest patient recorded in the literature with a transomental hernia through the greater omentum being 90 years [5]. The literature also suggests that most defects are on the right side [5], whereas as in our case series one such defect was on the right side and the other two being central and on the left side. These patients suffered from a chronic history of abdominal pain, with normal yet exhaustive investigations.

The aetiology of these defects varies from congenital, trauma or inflammatory causes [1]. However, with the increase of laparoscopic procedures being performed, iatrogenic defects in the greater omentum may become more prevalent in the future as a cause of intestinal obstruction. Early diagnostic laparoscopy perhaps can be advocated before exhausting the list of radiological procedures because it has been shown to be safe and associated with minimal morbidity [7].

The radiological investigations for such defects are difficult, as seen in our patients who had multiple negative investigations prior to laparoscopy. All our patients were found to have a defect in the greater omentum, whist the other type of omental herniations through the lesser omentum, in comparison, is more often detected pre-operatively with CT or barium studies [3-5]. Radiological investigations play a more useful role in the acute setting when patients present with bowel obstruction.

We wish to highlight the importance of looking for such defects in the setting of chronic abdominal pain, which has not been previously reported. The omentum is a viscus in its own right, and hence, at diagnostic laparoscopy, it should be inspected as a discrete entity, especially in the absence of any other pathology. It is also advisable that the greater omentum should be dealt carefully during other laparoscopic procedures to minimise trauma and creation of TOD, which can lead to bowel herniation and subsequent obstruction. Our review of the literature shows mainly cases from the radiological aspect during acute situations with patients presenting with bowel obstruction. To our knowledge, there is no literature discussing the role of the transomental defect in an elective setting prior to these patients possibly being admitted in the future as an emergency with transomental herniations.

## Abbreviations

CT: Computed Tomography
MRCP: Magnetic Resonance Cholangiopancreatography
MRI: Magnetic Resonance Imaging
OGD: Oesophago-Gastro-Duodenoscopy
TOD: Transomental Defect
USS: Ultrasound
